# Chest CT accuracy in the diagnosis of SARS-CoV-2 infection: initial experience in a cancer center

**DOI:** 10.1590/0100-3984.2020.0040

**Published:** 2020

**Authors:** Paula Nicole Vieira Pinto Barbosa, Almir Galvão Vieira Bitencourt, Gabriel Diaz de Miranda, Maria Fernanda Arruda Almeida, Rubens Chojniak

**Affiliations:** 1 Department of Imaging, A.C.Camargo Cancer Center, São Paulo, SP, Brazil.

**Keywords:** Computed tomography, Severe acute respiratory syndrome, Coronavirus infections, Cancer, Tomografia computadorizada, Síndrome respiratória aguda grave, Infecções por coronavírus, Câncer

## Abstract

**Objective:**

To evaluate the accuracy of chest computed tomography (CT) in patients with suspected severe acute respiratory syndrome-related coronavirus-2 (SARS-CoV-2) infection at a cancer center.

**Materials and Methods:**

This retrospective single-center study selected 91 patients who had chest CT and real-time polymerase chain reaction (RT-PCR) test collected at the same day. CT results were classified in negative, typical, indeterminate or atypical findings. Diagnostic accuracy, sensitivity and specificity were calculated for two different scenarios: in the first, only typical findings on CT were considered positive; in the second, both typical and indeterminate findings were considered positive.

**Results:**

Mean patients’ age was 58.2 years, most were male (60.4%) and had prior diagnosis of cancer (85.7%). CT showed typical findings in 28.6%, indeterminate findings in 24.2% and atypical findings in 26.4%. RT-PCR results were positive for SARS-CoV-2 in 27.5%. The sensitivity, specificity and accuracy in the first and second scenarios were respectively 64.0%, 84.8% and 79.1%, and 92.0%, 62.1% and 70.3%.

**Conclusion:**

CT has a high accuracy for the diagnosis of SARS-CoV-2 infection. Different interpretation criteria can provide either high sensitivity or high specificity. CT should be integrated as a triage test in resource-constrained environments during the pandemic to assist in the optimization of PCR-tests, isolation beds and intensive care units.

## Abstract (pt)

**Objetivo:**

Avaliar a acurácia da tomografia computadorizada (TC) de tórax em pacientes com suspeita de infecção por SARS-CoV-2 em um centro oncológico.

**Materiais e Métodos:**

Estudo retrospectivo e unicêntrico que selecionou 91 pacientes que realizaram TC de tórax e teste RT-PCR no mesmo dia. Os resultados da TC foram classificados em negativos, achados típicos, indeterminados ou atípicos. Acurácia diagnóstica, sensibilidade e especificidade foram calculadas para dois cenários: no primeiro, apenas TC com achados típicos foi considerada positiva; no segundo, achados típicos ou indeterminados foram considerados positivos.

**Resultados:**

A média de idade dos pacientes foi de 58,2 anos, sendo a maioria homens (60,4%) e com história de câncer prévio (85,7%). TC demonstrou achados típicos em 28,6%, indeterminados em 24,2% e atípicos em 26,4%. Resultados da RT-PCR foram positivos para SARS-CoV-2 em 27,5%. Sensibilidade, especificidade e acurácia no primeiro e segundo cenários foram, respectivamente, de 64,0%, 84,8% e 79,1%, e 92,0%, 62,1% e 70,3%.

**Conclusão:**

A TC tem alta acurácia para o diagnóstico de infecção por SARS-CoV-2. Diferentes critérios de interpretação fornecem maior sensibilidade ou especificidade. A TC deve ser integrada como um teste de triagem em ambientes com recursos limitados durante a pandemia, para ajudar na otimização da utilização de testes de PCR, leitos de isolamento e unidades de terapia intensiva.

## INTRODUCTION

The pandemic caused by the new coronavirus (SARS-CoV-2, severe acute respiratory syndrome-related coronavirus 2) has generated increasing demand for health services and there are still many uncertainties related to its management. The disease caused by this virus (COVID-19, coronavirus disease-19) is highly transmissible, but with low lethality (1-3.5%). However, it is estimated that 15-20% of infected people can develop severe pneumonia and 5-10% may need intensive care. Elderly patients with comorbidities are at higher risk of death from the disease. The current gold-standard for the etiological diagnosis of SARS-CoV-2 infection is reverse transcription polymerase chain reaction (RT-PCR) on respiratory tract specimens^(1,2)^.

Several studies have demonstrated the imaging aspects of this disease, with an emphasis on high-resolution computed tomography (CT) of the chest^(3-18)^. This method is not yet routinely recommended for screening or initial diagnosis of COVID-19, but it can be useful in assessing the severity of the disease, response to treatment, presence of complications and differential diagnoses^(19)^. Few studies have evaluated the diagnostic accuracy of chest CT in screening patients with suspected COVID-19^(11,17,18)^. This analysis is important due to the presence of other endemic and seasonal viruses, especially in scenarios where there is little availability of the serological test (PCR) or delay in providing the results.

Cancer patients appear to have a higher risk of developing COVID-19 as well as a worse prognosis of the disease when compared to non-cancer patients^(20)^. CT findings could be associated with a higher risk of developing severe events in cancer patients^(21)^. These patients are also at increased risk for the development of other pulmonary complications related to the disease itself or its treatment, such as opportunistic infections, thromboembolism, drug toxicity, actinic pneumonitis, among others^(22,23)^. These complications may represent differential diagnoses or be associated with SARS-CoV-2 infection in cancer patients.

The aim of this study was to evaluate the accuracy of chest CT in patients with clinical suspicion of COVID-19 at a cancer center.

## MATERIALS AND METHODS

This retrospective, observational, single-center study was approved by the National Commission for Research Ethics and informed consent was waived. We selected all patients who had chest CT due to suspected SARS-CoV-2 infection and RT-PCR test collected at the same day between February and March 2020 at a cancer center.

**Figure 1:**
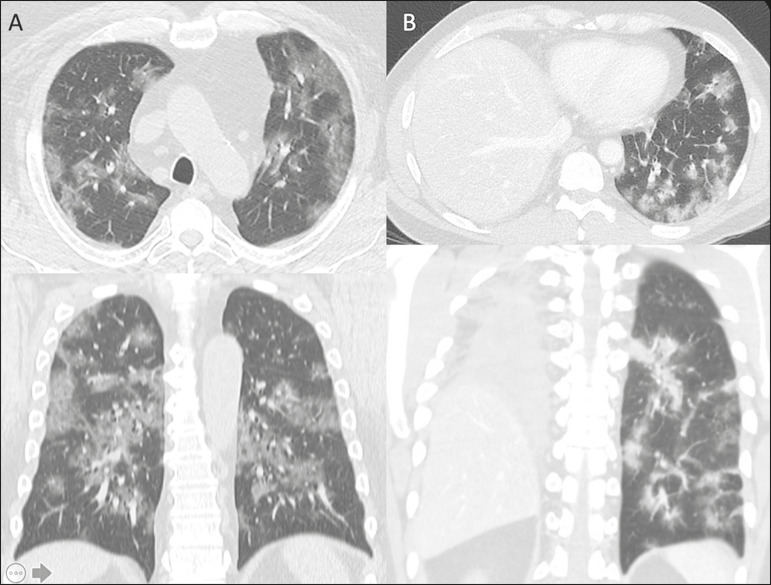
Examples of typical findings for COVID-19 on chest CT. **A:** Multifocal bilateral ground-glass opacities with typical distribution. **B:** Multifocal ground-glass opacities in a peripheral and lower distribution in the left lung in a patient with prior right pneumonectomy for lung cancer. Both cases had positive RT-PCR test for SARS-CoV-2 (true-positive results).

Clinical variables included age, gender, presence and type of neoplasia, time of onset of symptoms, signs and symptoms at admission and serological tests. Chest CT exams were reviewed by two experienced radiologists and imaging findings were classified, according to the consensus statement on reporting chest CT findings related to COVID-19 issued by the Society of Thoracic Radiology, the American College of Radiology, and the Radiology Society of North America^(24)^ in:

**Typical** - Multifocal ground-glass opacities with round morphology in a peripheral, posterior, and diffuse or lower lung zone distribution, with or without consolidation, visible intralobar lines (“crazy paving”), halo sign and reversed halo sign (Figure 1).

**Figure 2:**
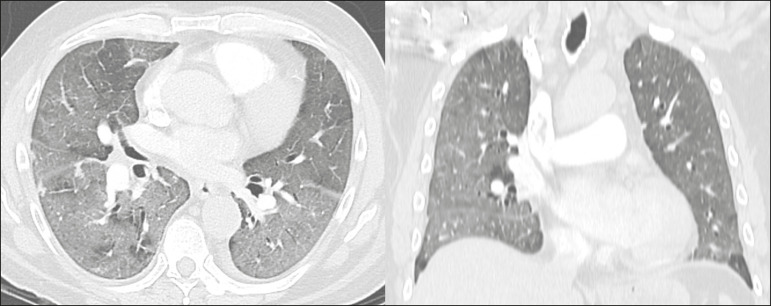
Example of indeterminate findings for COVID-19 on chest CT, showing bilateral diffuse ground-glass opacities with atypical distribution. RT-PCR test was positive for rinovirus.

**Indeterminate** - Nonspecific imaging features that do not match the typical features, such as ground-glass opacities with diffuse or central distribution, not rounded or unilateral (Figure 2).

**Figure 3:**
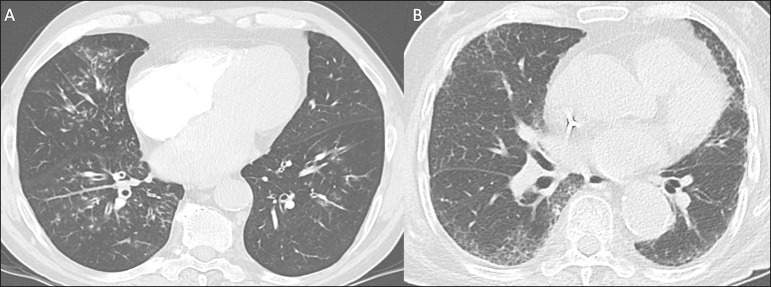
Examples of atypical findings for COVID-19 on chest CT. **A:** Small nodules with centrilobular “tree-in-bud” distribution in the right lung; RT-PCR test was positive for enterovirus. **B:** Typical findings of usual interstitial pneumonia in a patient with multiple myeloma.

**Atypical** - Isolated lobar or segmental consolidation without ground-glass opacities, discrete small nodules with centrilobular “tree-in-bud” distribution, cavitation, isolated pleural effusion, or mediastinal lymph node enlargement (Figure 3).

**Negative** - No significant findings on chest CT.

Frequencies and percentages were used to describe categorical variables, and mean, median, interquartile range (IQR) and range were used to describe continuous variables. Diagnostic accuracy, sensitivity, specificity, positive predictive value (PPV) and negative predictive value (NPV) were calculated for two different scenarios. In the first scenario, only chest CT with typical findings were considered positive, while in the second scenario, chest CT with typical or indeterminate findings were considered positive. The RT-PCR test was considered as the gold-standard. Proportions were provided, along with binomial exact 95% confidence intervals (CIs), using the “exact” Clopper-Pearson CIs for sensitivity and specificity, and the standard logit CIs for PPV and NPV^(25)^. We compared sensitivity and specificity between both scenarios using the McNemar test^(26)^. *P* values less than 0.05 were considered to represent statistically significant differences. All analyses were performed using SPSS software, version 20.0 (IBM Corp., Armonk, NY, USA).

## RESULTS

From 136 chest CT exams performed due to clinical suspicion of COVID-19 in the study period, 91 patients (66.9%) had RT-PCR results available and were included in the study. Most patients were male (n = 55; 60.4%). Mean age was 58.2 years (median, 61 years; IQR, 51-68 years; range, 19-88 years), including 47 (51.6%) patients with more than 60 years. Most common symptoms were cough reported by 55 patients (60.4%), fever in 34 (37.4%) and dyspnea in 36 (39.6%). The mean of referred symptoms duration was 6.0 days (median, 5 days; IQR, 1-7 days; range, 1-30 days), including 30 (33.0%) patients with 0-2 days, 15 (16.5%) with 3-5 days and 18 (19.8%) with 6-7 days.

Most patients (n = 78; 85.7%) had prior diagnosis of cancer, being the most common hematologic malignancies (n = 21; 23.1%), breast cancer (n = 13; 14.3%) and lung cancer (n = 8; 8.8%). At least, 25 patients (27.5%) were undergoing oncologic treatment (chemotherapy, immunotherapy or radiotherapy) in the moment of the exam. Other comorbidities were present in 17 patients (18.7%), the more common were cardiopathy and pneumopathy in 6 cases (6.6%) each.

Chest CT showed typical findings in 26 patients (28.6%), indeterminate findings in 22 (24.2%), atypical findings in 24 (26.4%) and was negative in 19 (20.9%). RT-PCR results were negative in 55 cases (60.4%), positive for SARS-CoV-2 in 25 (27.5%) and positive for other viruses in 11 (12.1%).

In the first scenario, considering only chest CT with typical findings as positive, the sensitivity, specificity, PPV, NPV and accuracy were respectively 64.0%, 84.8%, 61.5%, 86.2% and 79.1% (Table 1). In the second scenario, considering chest CT with typical or indeterminate findings as positive, the sensitivity, specificity, PPV, NPV and accuracy were respectively 92.0%, 62.1%, 47.9%, 95.3% and 70.3% (Table 2). The sensitivity of chest CT was higher in the second scenario (92.0% vs. 64.0%; *p* = 0.016) and the specificity was higher in the first scenario (84.8% vs. 62.1%; *p* < 0.001).

**Table 1:** Chest CT accuracy for SARS-CoV-2 infection considering only typical findings as positive.

Chest CT	RT-PCR		
	Positive	Negative	Total
Positive	16	10	26
Negative	9	56	65
Total	25	66	91

Sensitivity: 16/25 (64.0%); 95% CI: 42.5-82.0%. Specificity: 56/66 (84.8%); 95% CI: 73.9-92.5%. PPV: 16/26 (61.5%); 95% CI: 45.7-75.3%. NPV: 56/65 (86.2%); 95% CI: 78.5-91.4%. Accuracy: 72/91 (79.1%); 95% CI: 69.3-86.9%.

**Table 2:** Chest CT accuracy for SARS-CoV-2 infection considering typical and indeterminate findings as positive.

Chest CT	RT-PCR		
	Positive	Negative	Total
Positive	23	25	48
Negative	2	41	43
Total	25	66	91

Sensitivity: 23/25 (92.0%); 95% CI: 74.0-99.0%. Specificity: 41/66 (62.1%); 95% CI: 49.3-73.8%. PPV: 23/48 (47.9%); 95%. CI: 39.8-56.1%. NPV: 41/43 (95.3%); 95% CI: 84.3-98.7%. Accuracy: 64/91 (70.3%); 95% CI: 59.8-79.5%.

## DISCUSSION

The results of the present study show that chest CT has a good performance for the diagnosis of SARS-CoV-2 infection patients at a cancer center. We classified the results of chest CT scans according to findings already described for the interpretation of studies in patients with suspected COVID-19. We studied two scenarios for the CT interpretation: in the first one, we considered positive for COVID-19 only chest CT with typical findings and, in the second one, we also considered positive the CTs with findings described as indeterminate. This information can be useful in a triage situation when CT scans are available but access to the RT-PCR test is limited, the results are time consuming and there is a shortage of hospital beds and isolation units. It is interesting to note that the first scenario showed high specificity, which gives confidence to select patients with positive results as those who should benefit from investigative or intervention measures, because there will be few false-positive results. The second scenario showed high sensitivity, which provides confidence to select patients with negative CT results as those in which other investigative or intervention measures can be avoided or optimized, such as RT-PCR test, isolation and hospitalization, because false negative CT results are rare.

We therefore believe that our data add clinical evidence that allow the CT triage recommendation, as an alternative indication, in resource-constrained environment for patients with suspicion for COVID-19 infections. Recently, a Fleishner Society statement suggested that chest CT could be indicated for medical triage of patients with suspected COVID-19 who present with moderate-severe clinical features and a high pre-test probability of disease, especially when rapid point-of-care COVID-19 tests are not available^(19)^.

Prior studies found a high sensitivity (97%) and low to moderate specificity (25-56%), however they did not define the criteria used to classify chest CT findings as positive in that study^(11,18)^. On the other hand, Bai et al. demonstrated that CT has a high specificity to differentiate COVID-19 from other viral pneumonias, with moderate sensitivity^(17)^. These findings suggest that different criteria used to evaluate CT findings can achieve different diagnostic accuracy results.

It is also important to consider that the imaging findings also depends on when infected patients are imaged^(8,12,16)^. More than half of patients with less than two days from the symptoms’ onset could have a negative CT^(27)^. In our sample, 30 patients had CT scans performed less than two days from the symptoms’ onset and only two of them had false-negative results.

This study has some limitations related to the small sample size and short follow-up. RT-PCR test, which was used as gold-standard in this study, is believed to have high specificity but sensitivity of 60-70% to diagnose SARS-CoV-2 infection^(10,11)^. It is known that RT-PCR has potential vulnerabilities related to preanalytical issues such as inadequate procedures for collection, handling, transport and storage of the swabs, as well as analytical problems, including testing outside the diagnostic window and other specific technical issues^(28)^. Prior studies demonstrated that COVID-19 patients may show very early CT changes even before positive RT-PCR^(11,29)^. Probably, the most efficient strategy for diagnosing COVID-19 in suspected patients should combine clinical and epidemiologic suspicion with RT-PCR test and chest CT findings, and repeated RT-PCR should be collected in patients with initially negative results and high clinical and imaging likelihood of having COVID-19.

In conclusion, CT has a high diagnostic accuracy for the diagnosis of SARS-CoV-2 infection and should be integrated as a triage test in resource-constrained environments during the pandemic to assist in the optimization of PCR-tests, isolation beds and intensive care units.
